# De novo transcriptome assembly and annotation of parthenogenetic lizard *Darevskia unisexualis* and its parental ancestors *Darevskia valentini* and *Darevskia raddei nairensis*

**DOI:** 10.1016/j.dib.2021.107685

**Published:** 2021-12-06

**Authors:** Sergei S. Ryakhovsky, Victoria A. Dikaya, Vitaly I. Korchagin, Andrey A. Vergun, Lavrentii G. Danilov, Sofia D. Ochkalova, Anastasiya E. Girnyk, Daria V. Zhernakova, Marine S. Arakelyan, Vladimir B. Brukhin, Aleksey S. Komissarov, Alexey P. Ryskov

**Affiliations:** a Laboratory of Genome Organization, Institute of Gene Biology of the Russian Academy of Sciences, Vavilova Str., 34/5, Moscow 119334, Russia; b Applied Genomics Laboratory, SCAMT Institute, ITMO University, Lomonosova 9 Str., Saint Petersburg 197101, Russia; c Department of Biochemistry, Molecular biology and Genetics, Moscow Pedagogical State University, 1/1 M.Pirogovskaya Str., Moscow 119991, Russia; d Department of Genetics and Biotechnology, St. Petersburg State University, 7/9 Universitetskaya Nab., St. Petersburg 199034, Russia; e Laboratory of Genomic Diversity, Center for Computer Technologies, ITMO University, Kronverksky Ave. 49, St. Petersburg 197101, Russia; f Faculty of Biology, Yerevan State University, 1 Alex Manoogian, 0025, Yerevan , Armenia; g Plant Genomics Laboratory, SCAMT Institute, ITMO University, Saint Petersburg 197101, Russia

**Keywords:** *Darevskia* lizards, Parthenogenesis, Transcriptome analysis, Ovaries, AMP

## Abstract

*Darevskia* rock lizards include 29 sexual and seven parthenogenetic species of hybrid origin distributed in the Caucasus. All seven parthenogenetic species of the genus *Darevskia* were formed as a result of interspecific hybridization of only four sexual species. It remains unknown what are the main advantages of interspecific hybridization along with switching on parthenogenetic reproduction in evolution of reptiles. Data on whole transcriptome sequencing of parthenogens and their parental ancestors can provide value impact in solving this problem. Here we have sequenced ovary tissue transcriptomes from unisexual parthenogenetic lizard *D. unisexualis* and its parental bisexual ancestors to facilitate the subsequent annotation and to obtain the collinear characteristics for comparison with other lizard species. Here we report generated RNAseq data from total mRNA of ovary tissues of *D. unisexualis, D. valentini* and *D. raddei* with 58932755, 51634041 and 62788216 reads. Obtained RNA reads were assembled by Trinity assembler and 95141, 62123, 61836 contigs were identified with N50 values of 2409, 2801 and 2827 respectively. For further analysis top Gene Ontology terms were annotated for all species and transcript number was calculated. The raw data were deposited in the NCBI SRA database (BioProject PRJNA773939). The assemblies are available in Mendeley Data and can be accessed via doi:10.17632/rtd8cx7zc3.1.

## Specifications Table

**Table:** 

Subject	Biology
Specific subject area	Transcriptomics
Type of data	Transcriptome assemblies, raw sequences
How data were acquired	Ovary RNA from three lizard species were isolated and used for sequencing by the Macrogen Inc. (Korea)
Data format	Analyzed, Raw
Parameters for data collection	Data collection contains raw transcriptome data for ovary tissues of three lizard species: unisexual (parthenogenetic) *D. unisexualis* and parental bisexual *D. valentini* and *D. raddei nairensis*
Description of data collection	Data collection includes total Illumina HiSeq2500 generated transcriptome reads, transcripts, TRINITY contigs, predicted proteins, and ORFs.
Data source location	All lizards were collected from Armenia populations. *D. unisexualis* from the Hrazdan population (40.503493 N 44.748097 E) *D. r. nairensis* from Vahramaberd population (40.844394 N, 43.755720 E) *D. valentini* from Sepasar population (41.027492 N, 43.816634 E)
Data accessibility	Raw data - BioProject PRJNA773939 in NCBI SRA database. Trinity assemblies - doi:https://doi.org/10.17632/rtd8cx7zc3.1 in Mendeley Data

## Value of the Data

• Data provides information about the first assembled ovary transcriptomes of three genetically related Darevskia lizards species and information about their genes and proteins.
• This data may benefit evolutionary biologists because it shows genetic differences between unisexual (parthenogenetic) and bisexual parental lizards.
• The data may provide insight into the genetic underpinning of parthenogenetic reproduction and can be used in further study of these genes.

## 1 Data Description

**Fig. 1:**
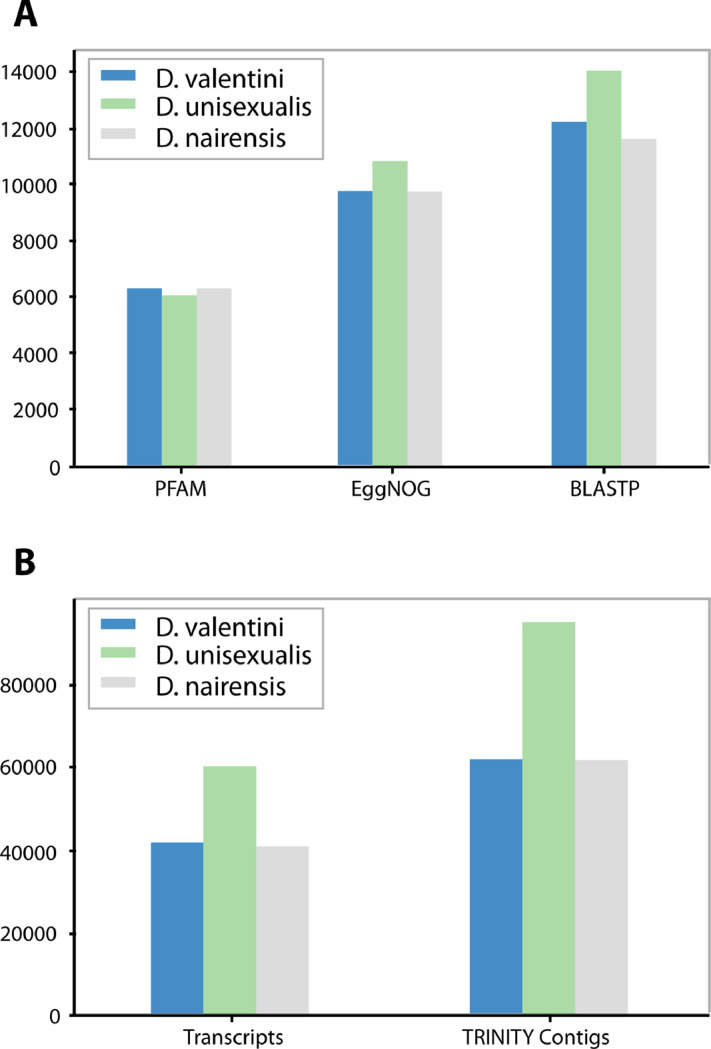
Structural characteristics of three transcriptomes. A number of annotated proteins (A), and TRINITY contigs, and transcripts (B).

**Fig. 2:**
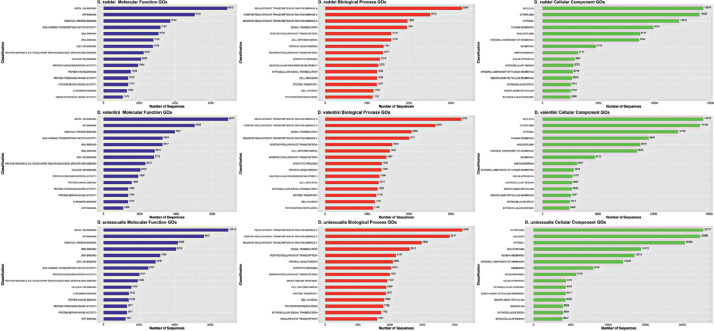
Distribution of the top 10 GO terms for each of the three species.

**Fig. 3:**
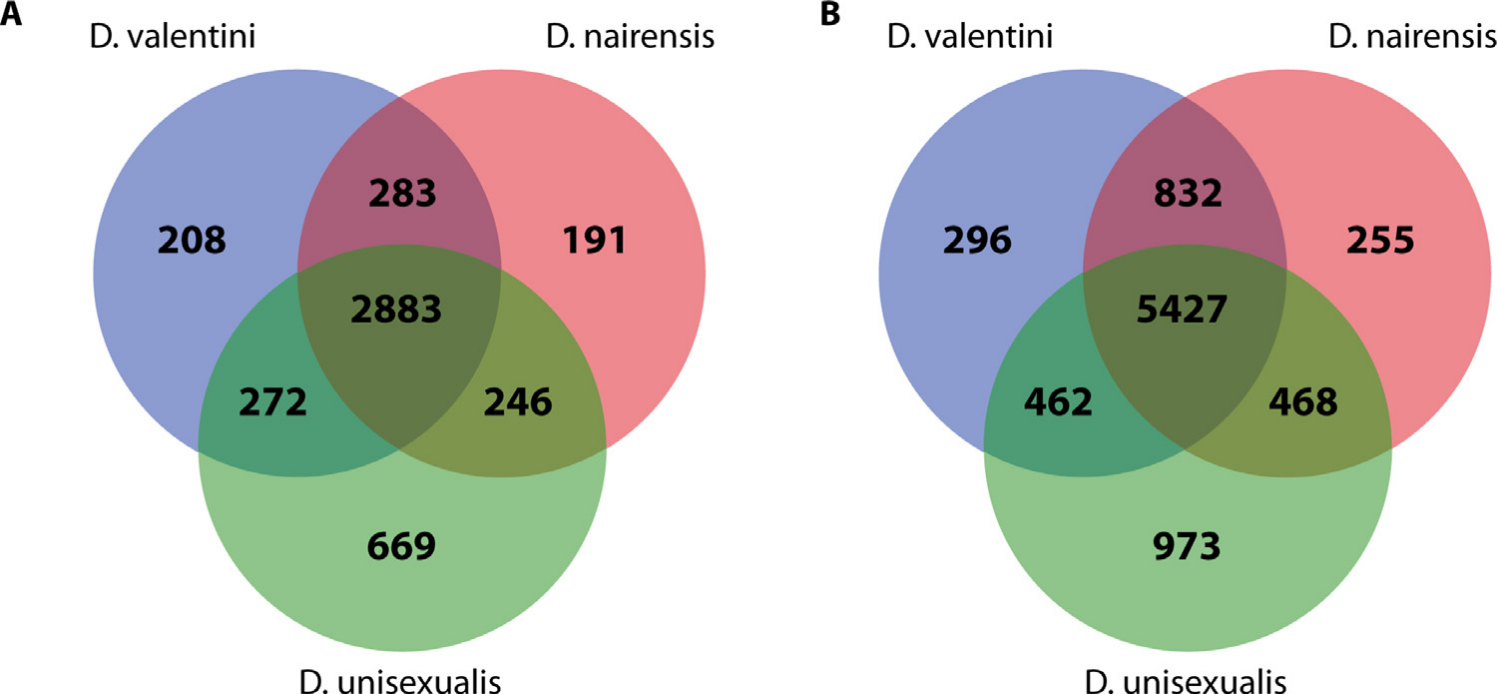
Venn diagrams showing overlapping hits for three species (A) and overlapping of EggNOG genes (B).

Ovary RNA from three individuals of each species was pooled together and used to prepare the three cDNA libraries: *D. unisexualis*, *D. raddei nairensis, D. valentini*. Table 1 shows the total number of bases, reads, GC (%), Q20 (%), and Q30 (%) that were calculated for the three samples. The characteristics of assembled transcriptome sequences are presented in Table 2. Structural characteristics of three transcriptomes are shown in Fig. 1. Obtained Trinity assemblies contain 60132 transcripts for *D. unisexualis*, 41680 for *D. valentini,* and 413664 for *D. r. nairensis*. TransDecoder peptide output was used for BLASTP, Pfam, and EggNOG search (Fig. 1A, Supplementary 1). BLASTP v. 2.9.0+ revealed 14049, 12331, and 11865 proteins for *D. unisexualis, D. valentini,* and *D. nairensis* respectively (Fig. 1A). Parthenogenetic species *D. unisexualis* showed greater TRINITY contigs (> 81.4% and > 87.2%) and transcripts (> 44.3% and > 45.4%) numbers than *D. valentini* and *D. r. raddei* respectively (Fig. 1B). The *D. unisexualis* showed more hits for each of the searching instruments. Top 10 GO terms taken from all GO terms datasets and distribution graphs are presented in Fig. 2 (Supplementary 2)*.* The biggest number of annotated genes and the most annotated category was a cellular component, biological processes were less annotated. In the molecular functions category, most genes were related to binding. The most highly enriched genes in biological processes were related to the regulation of transcription of RNA polymerase II. It was found that in cellular components over-represented molecules were the nucleus and cytoplasm origin. In total, 38844, 38756, 63219 transcripts with GO terms were annotated in Table 3 for *D. valentini, D.raddei, and D. unisexualis* respectively. The summary of Trinotate shows a more prevalent number of annotated transcripts with GO in *D. unisexualis* than in *D. valentini* and *D. raddei nairensis* (> 62.8% and > 63.1%). The total number of GO in the parthenogenetic sample exceeds *D. valentini* and *D. raddei nairensis* on 58.9% and 60% respectively (Table 3). The final TransDecoder stats are presented in Table 4. The overall number of ORFs in D. unisexualis was 45.7% more than in bisexual parental samples, according to the TransDecoder results.. The analysis of common and unique genes on Venn diagrams (Fig. 3A, B) displays that *D. unisexualis* has more unique genes in BLASTP (> 221.6% and > 250%) and EggNOG (> 228.7% and > 281.6%) than *D. valentini* and *D. nairensis* respectively (Supplementary 4). The antimicrobial peptides have been searched in *D. valentini, D. nairensis,* and *D. unisexualis* with 59, 81, and 70 possible matches respectively. These sequences were found in 29, 34, and 36 transcripts. The antimicrobial peptides detected have antibacterial activity against Gram+, Gram- as well as against fungi (Supplementary 5). The raw RNA sequence reads for each lizard are available in the NCBI SRA database (PRJNA773939). The assembled transcripts are available in the Mendeley data (doi:https://doi.org/10.17632/rtd8cx7zc3.1).

**Table 1:** Statistics of the RNA-seq generated from three lizards.

Species	Total reads	Total bases	Q20 bases^a^	Q30 bases^b^	GC content
*D. unisexualis*	58.932755 M	17,313222 G	97.98%	94.34%	48.05%
*D. valentini*	51.634041 M	15,593480 G	97.65%	93.35%	46.59%
*D. r. nairensis*	62.788216 M	18,962041 G	97.13%	92.33%	46.77%

a Q20 - ratio of bases with probability of containing no more than one error in 100 bases.

b Q30 - ratio of bases with probability of containing no more than one error in 1,000 bases.

**Table 2:** Summary characteristics of transcriptome sequence assembly of all three samples data.

	*D. raddei nairensis*	*D. valentini*	*D. unisexualis*
# contigs (>= 0 bp)	122746	126141	228862
# contigs (>= 1000 bp)	39398	39145	57984
# contigs (>= 5000 bp)	3517	3533	2447
# contigs (>= 10000 bp)	134	118	8
# contigs (>= 25000 bp)	0	0	0
# contigs (>= 50000 bp)	0	0	0
Total length (>= 0 bp)	139903614	140424203	204321108
Total length (>= 1000 bp)	105256962	104453618	138481341
Total length (>= 5000 bp)	22937215	22851989	14738047
Total length (>= 10000 bp)	1524044	1315073	90998
Total length (>= 25000 bp)	0	0	0
Total length (>= 50000 bp)	0	0	0
# contigs	61836	62123	95141
Largest contig	16187	14170	12181
Total length	121008445	120525010	164367910
GC (%)	46,23	46,19	46,06
N50	2827	2801	2409
N75	1567	1558	1378
L50	13677	13716	22778
L75	27887	27951	45070
# N's per 100 kbp	0.0	0.0	0.0

**Table 3:** Summary of Trinotate/Gene Ontologies.

Species	Total transcripts with GO	Total transcripts with only one GO	Total transcripts with multiple GO	Total GO in the file	Total unique GO in the file
*D. valentini*	38844	1241	37603	553827	16934
*D. nairensis*	38756	1195	37561	550189	16885
*D. unisexualis*	63219	1796	61423	880200	17771

**Table 4:** Open Reading Frames (ORFs) prediction numbers using TransDecoder.

Species	Total	Complete	5-prime partial	3-prime partial	Internal
*D. valentini*	55816	34333	12663	3133	22136
*D. nairensis*	55808	34499	12893	3026	5327
*D. unisexualis*	81344	43408	22136	5390	10473

## 2 Experimental Design, Materials and Methods

### 2.1 Species sampling and tissues collection

Samples of *D. valentini, D. r. nairensis,* and *D. unisexualis* for transcriptome analysis were collected in Armenia in 2019, outside of the protected areas. Several adult lizards of female *D. unisexualis* from the Hrazdan population (40.503493 N 44.748097 E), females *D. r. nairensis* from the Vahramaberd population (40.844394 N, 43.755720 E), and females *D. valentini* from the Sepasar population (41.027492 N, 43.816634 E) were used to surgically extract ovary. Before dissecting out the organs, the animals were subjected to chloroform euthanasia. All tissue samples were stored in RNAlater® reagent at −20°C according to the manufacturer's recommended protocol (Qiagen Inc.) until they were shipped to Macrogen Inc. (Korea) for RNA extraction and further transcriptome preparation.

### 2.2 RNA sequencing and raw data quality control

Total RNA was isolated from an organ/tissue using standard Trizol Tissue RNA Extraction protocol (Standard protocol for QIAzol Lysis Reagent, Qiagen). RNA RIN scores ranged from 6.4 to 6.7. Ovary RNA from three individuals of each species was pooled together and used to prepare the three cDNA libraries: *D. unisexualis*, *D. r. nairensis, D. valentini*. Inside the procedure was a cleanup on a carrier with polyT and random primers from TruSeq Stranded mRNA kit were used for preparation cDNA. The paired-end sequencing libraries were prepared by random fragmentation of the cDNA samples into 350-500 bp fragments, followed by 5′ and 3′ adapter ligation using TruSeq RNA Sample Prep Kit v2 (Illumina Inc.) according to TruSeq RNA Sample Preparation Guide (Version 2, Part #15026495 Rev.F).

Sequencing of transcriptome libraries was performed on Illumina HiSeq2500 with a mean read length of 101 bp. The Illumina Hiseq generated raw sequencing data utilizing HiSeq Control Software v2.2 for system control and base calling through an integrated primary analysis software. The BCL (base calls) binaries were converted into FASTQ by the Illumina package bcl2fastq (v1.8.4) [1] (RRID:SCR_015058). Raw transcriptome data were trimmed by Trimmomatic v0.39 [2] to remove adapters. Optical duplicates from reads were removed by the rmdup tool [3]. Raw transcriptomes contained 58932755, 51634041, and 62788216 reads for *D. unisexualis, D. valentini* and *D. r. nairensis* with GC content of 48.05%, 46.59%, and 46.77% respectively. Filtered reads quality was estimated by FastQC v0.11.9 [4] and became prepared for assembling.

### 2.3 Transcriptome annotation and assembly

Reads obtained after trimming and quality estimating by FastQC and Seq2fun pipeline [5] were assembled using Trinity v2.1.1 [6]. Transcriptome assembly with Trinity can be divided into several parts: searching and calculating k-mers, assembling contigs from k-mers, clustering contigs into components. For Trinity assembler, the default parameters were taken, where the minimum contig length value was 200, k-mer size was 25. TransDecoder v5.5.0 [7] program was used to predict translated proteins and ORFs (open reading frames) from assembled transcripts with at least 100 amino acids length. NCBI-blast-2.9.0+ [8] was used for homology search and protein domain identification on TransDecoder predicted proteins with such parameters as e-value < 1e-5 and percentage of similarity > 95%.

The total number of bases, reads, GC (%), Q20 (%), and Q30 (%) were calculated for the three samples (Table 1). Obtained protein sequences from TransDecoder were cross-referenced with the Gene Ontology (GO) [9,10] database using the EggNog v2.0.1 [11] tool. This tool provides functional information in the context of structure, molecular functions, the biological process of query sequences, search matches, and performs them as GO terms. Top GO terms were determined and visualized using the Trinotate package in the TransPi [12] pipeline. PFAM [13] and BLASTP searches were also performed by the TransPi pipeline with OnlyAnn (only annotation) option. This mode used such databases as Swissprot, Uniprot custom database (available under request), and Pfam.

### 2.4 AMPs identification

To identify antimicrobial peptides (AMP) in the transcriptome, we blasted the assembled transcripts against the known AMPs from the DRAMP 3.0 database (Data Repository of Antimicrobial Peptides) [14] using BLAST-2.2.26+ [15] with the similarity cutoff of 70%.

## Ethics Statement

All individuals were hand-caught; alive-animal handling procedures were approved by Yerevan State University according to the ethical guidelines, capture permit Code 5/22.1/51043 was issued by the Ministry of Nature Protection of the Republic of Armenia for scientific studies. The study was approved by the Ethics Committee of the Moscow State University (Permit Number: 24–01) and conducted strictly according to ethical principles and scientific standards.

## CRediT authorship contribution statement

**Sergei S. Ryakhovsky:** Formal analysis, Writing – original draft, Writing – review & editing. **Victoria A. Dikaya:** Formal analysis. **Vitaly I. Korchagin:** Data curation. **Andrey A. Vergun:** Writing – original draft, Writing – review & editing, Data curation, Methodology. **Lavrentii G. Danilov:** Formal analysis, Writing – original draft, Writing – review & editing. **Sofia D. Ochkalova:** . **Anastasiya E. Girnyk:** Data curation. **Daria V. Zhernakova:** Conceptualization, Visualization. **Marine S. Arakelyan:** Methodology, Supervision. **Vladimir B. Brukhin:** Writing – original draft, Writing – review & editing. **Aleksey S. Komissarov:** Project administration, Conceptualization, Visualization, Data curation, Writing – original draft, Writing – review & editing, Supervision. **Alexey P. Ryskov:** Project administration, Conceptualization, Writing – original draft, Writing – review & editing, Supervision, Funding acquisition.

## Declaration of Competing Interest

All authors have read and approved the final manuscript. Consent for publication: Not applicable. The authors declare that they have no competing interests.

## Appendix Supplementary materials

Image, application 1

## Footnotes

Supplementary material associated with this article can be found in the online version at doi:10.1016/j.dib.2021.107685.
